# Perceptions About Mindfulness and Text Messaging for Smoking Cessation in Vietnam: Results From a Qualitative Study

**DOI:** 10.2196/17337

**Published:** 2020-06-24

**Authors:** Vuong Van Do, Claire Adams Spears, Hoang Van Minh, Jidong Huang, Pamela Buffington Redmon, Nguyen Xuan Long, Michael Paul Eriksen

**Affiliations:** 1 Center for Population Health Sciences Hanoi University of Public Health Hanoi Vietnam; 2 School of Public Health Georgia State University Atlanta, GA United States; 3 Emory Global Health Institute Emory University Atlanta, GA United States; 4 Vietnam Association of Psychology Hanoi Vietnam

**Keywords:** mHealth, mobile health, text messages, smoking, smoking cessation, mobile phone

## Abstract

**Background:**

With 15.6 million smokers, Vietnam is one of the top 10 largest cigarette-consuming countries in the world. Unfortunately, smoking cessation programs are still scarce in Vietnam. Mindfulness-based and text messaging–based interventions have been increasingly used in smoking cessation studies in developed countries, with promising results. Given the exponential growth of mobile phone usage in Vietnam in recent years, mobile health interventions could be a potential strategy to increase smoking cessation in Vietnam. However, substantial cultural adaptations are needed to optimize the effectiveness of these interventions among Vietnamese smokers.

**Objective:**

This study aims to involve qualitative research to inform the development of a mindfulness-based text messaging smoking cessation intervention for Vietnamese smokers.

**Methods:**

A total of 10 focus groups were conducted with 71 Vietnamese male smokers aged between 18 and 65 years (5-9 participants per focus group). Overall, 5 focus groups were conducted with smokers who had the intention to quit (ie, preparation stage of change in the transtheoretical model), and 5 focus groups were conducted with smokers who did not have the intention to quit (contemplation or precontemplation stage). The focus groups were audio recorded, transcribed verbatim, and analyzed using NVivo 12 software (QSR International).

**Results:**

The major themes included smoking triggers, barriers and facilitators for quitting, the perceptions of text messaging and mindfulness approaches for smoking cessation, and suggestions for the development of a text messaging–based smoking cessation program. Common smoking triggers included stress, difficulties concentrating, and fatigue. Frequently encountering other people who were smoking was a common barrier to quitting. However, participants indicated that concerns about the harmful effects of smoking on themselves and their wives and children, and encouragement from family members could motivate them to quit. The participants preferred diverse message content, including information about the consequences of smoking, encouragement to quit, and tips to cope with cravings. They suggested that text messages be clear and concise and use familiar language. Most smokers perceived that mindfulness training could be useful for smoking cessation. However, some suggested that videos or in-person training may also be needed to supplement teaching mindfulness through text messages.

**Conclusions:**

This study provides important insights to inform the development of a text messaging–based smoking cessation program that incorporates mindfulness for Vietnamese male smokers. The results could also be useful for informing similar programs in other low- and middle-income countries.

## Introduction

Smoking is the leading cause of preventable deaths worldwide and a major risk factor for most noncommunicable diseases [1,2]. It has been estimated that smoking would cause 150 million deaths globally by 2030, and 80% of these deaths would occur in low- and middle-income countries [3]. Helping smokers to quit smoking is considered by many to be the only practical way to avoid a substantial proportion of tobacco-related deaths worldwide before 2050 [4].

With 15.6 million smokers [5], Vietnam (a lower-middle-income country in Southeast Asia) is one of the top 10 largest cigarette-consuming countries worldwide [6]. Most Vietnamese smokers are men, with a prevalence of smoking of 45.3% among men and 1.1% among women in 2015 [5]. Smoking imposes a tremendous health and economic burden on Vietnam [7,8]. Over 66,000 deaths in Vietnam were attributed to major tobacco-related diseases in 2013 alone [9]. Unfortunately, smoking cessation programs are still scarce [10], even though Vietnam has ratified the World Health Organization Framework Convention on Tobacco Control since 2004. Smoking cessation programs that incorporate evidence-based strategies and are culturally appropriate and cost-effective are urgently needed in Vietnam.

Mobile health (mHealth) interventions have tremendous potential for cost-effective dissemination of smoking cessation interventions. In particular, text messaging programs enable the provision of tailored advice and support, can be delivered at a relatively low cost, and are scalable for a wide public health impact [11]. Recent systematic reviews show that the quit rates among smokers receiving text messaging–based interventions were consistently higher than those in control groups, with odds ratios ranging from 1.35 to 2.89 [11-14]. However, the majority of these published studies were conducted in high-income countries and none in Vietnam. In 2018, there were 147 mobile phone subscriptions per 100 people in Vietnam, which exceeded that of high-income countries (126 per 100 people) [15]. Moreover, a recent study found that 72% of Vietnamese smokers with the intention to quit were willing to use and pay for smoking cessation support via text messages if available [16]. Thus, text messaging appears to be an acceptable modality for smoking cessation in Vietnam. However, formative research is needed before developing an intervention to ensure that the language, content, and format are culturally appropriate.

Mindfulness training could be an important culturally relevant strategy that has not yet been examined in smoking cessation interventions in Vietnam. Mindfulness is defined as purposeful, present-focused attention, with an attitude of acceptance and nonjudgment [17,18]. Mindfulness originated in Buddhist traditions and is central to the Theravada Buddhist tradition and Mahayana (Zen) schools of Vietnam [19]. Mindfulness-based interventions show promise for promoting smoking cessation in diverse populations in the United States [20]. These interventions teach smokers to notice and pay attention to their emotional states and their cravings with a nonjudgmental attitude. For example, rather than impulsively reacting to cravings by smoking, mindfulness can help smokers to consciously choose how to respond in healthier ways [21]. Mindfulness practices such as mindful breathing (focusing on one’s breath) do not require a high level of education or resources. Therefore, a text messaging program with the integration of mindfulness messages might be beneficial in low-resource settings.

Given that no published research has examined either mindfulness or mHealth for smoking cessation in Vietnam, this qualitative study sought to gain feedback from Vietnamese smokers to inform intervention development. Specifically, this study aimed to understand smoking triggers and barriers and facilitators for quitting smoking among Vietnamese male smokers. We also examined the perceptions of participants about mindfulness and text messaging strategies for smoking cessation, including questions about the content and frequency of text messages. The results of this study will be used to inform the development of a mindfulness-based mHealth smoking cessation program in Vietnam, and the findings might also be useful for guiding similar interventions in other low- and middle-income countries.

## Methods

### Design

A total of 10 focus groups were conducted among adult Vietnamese male smokers, half (5 focus groups) of which were conducted with smokers who intended to quit in the next 30 days (ie, in the *preparation* stage of change of the transtheoretical model [22]) and the rest with smokers who did not have intentions to quit in the next 30 days (in the *contemplation* or *precontemplation* stage; see details in Participants and Recruitment).

### Participants and Recruitment

Participants were contacted by recruiters (health staff at commune health stations) and were asked to complete the screening questions to determine their eligibility and stage of change. Individuals were eligible if they were Vietnamese men between the ages of 18 and 65 years and reported that they currently smoked cigarettes. Female smokers were not included in this study because only 1.1% of Vietnamese women are smokers, compared with 45.3% among men [5]. Smokers who reported intentions to quit within the next 30 days and had at least one 24-hour quit attempt in the past year were classified into the preparation stage [23]. Smokers who were either not interested in quitting or indicated an interest in quitting in the next 6 months (but not the next 30 days) were classified into the precontemplation and contemplation stages, respectively, [23].

### Data Collection and Procedures

Data collection occurred between March and April 2018. The quantitative survey questionnaires and the focus group moderator guide were developed by the research team in English and then translated to Vietnamese. The research team provided detailed information about the study and obtained written informed consent from all participants. Before participating in the group discussion, the participants completed a short survey on their sociodemographic background, smoking habits, cell phone usage (ie, type of cell phone, internet access, and texting habits), and level of nicotine dependence (Fagerström test for nicotine dependence; classified into four levels: low, low to moderate, moderate, and high [24]).

Each group discussion lasted between 90 and 120 minutes. The facilitator followed a semistructured interview guide that asked the participants about their smoking triggers; barriers and facilitators to quitting; level of interest in text messaging to help them quit smoking; suggestions for the content of the messages, including mindfulness-based messages; preferences for the structure and timing of the messages; and other ideas for making the program more helpful and user-friendly. Some technical terms were explained to the participants to ask for their suggestions, such as interactive messages (ie, users would interact with the SMS system by answering questions and receiving more information based on their answer) or keywords (users could send keywords such as “stress”, “crave”, or “slip” to get extra support for coping with stress, craving, or smoking lapses). The concept of mindfulness (translated as 
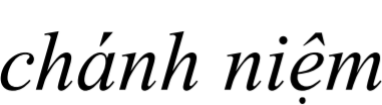
 in Vietnamese) was explained to the participants, and then the participants watched a short video about mindful breathing that led them into a 3-min mindful breathing practice. After the mindfulness practice, participants were asked about their understanding of mindfulness and their thoughts about incorporating mindfulness into smoking cessation treatment. All group discussions were audio recorded using a digital voice recorder. This study was approved by the institutional review board of Georgia State University (reference number H18030).

### Data Analysis

Descriptive statistics were applied for quantitative data analyses (ie, frequency and percentage for categorical variables and mean and range for continuous variables). With regard to qualitative data, all recordings were transcribed verbatim and then translated into English for coding. The transcripts were managed and coded using NVivo 12 software (QSR International). Data coding and analysis followed both inductive and deductive approaches [25,26]. A coding manual was first developed by 2 research team members, based on the interview guide and other recurring concepts in the focus group transcripts. The coding manual was refined through group discussions among all research team members. The remaining transcripts were then independently coded by 2 members who were bilingual in Vietnamese and English. During the coding process, regular meetings that included all research team members were held to resolve coding discrepancies, maintain coding consistency over time, and further refine the coding scheme as needed. Discrepancies were resolved through group discussions and consensus among all research team members.

## Results

### Characteristics of the Participants

A total of 71 adult male smokers participated in 10 focus groups, 5 of which were conducted among 37 smokers with the intention to quit, and the other 5 with 34 smokers who had no intention to quit. The demographic and background characteristics of these 71 participants are presented in Table 1. The mean age of participants was 42.2 years. Approximately 80% of the participants had a cell phone that could access the internet. Half of the participants reported that they often checked new messages immediately as they are delivered. More than 90% of the participants were daily smokers, and they smoked 13 cigarettes on average per day. Most participants had moderate-to-high levels of nicotine dependence based on the Fagerström test for nicotine dependence. Although smokers who were single were 3 times as likely to be in the contemplation or precontemplation stage, there were no substantial differences in sociodemographic or smoking-related characteristics between the participants in the preparation stage and the contemplation or precontemplation stage.

**Table 1:** Participant characteristics.

Characteristics		Preparation stage (n=37)	Contemplation or precontemplation stage (n=34)	Total (N=71)
Age (years), mean (range)		42.7 (19-56)	41.7 (18-64)	42.2 (18-64)
**Education, n (%)**				
	Less than secondary school	2 (5)	2 (6)	4 (6)
	Secondary school completed	9 (24)	9 (27)	18 (25)
	High school completed	23 (62)	19 (56)	42 (60)
	University/college	3 (8)	4 (12)	7 (10)
**Working status, n (%)**				
	Government employee	8 (22)	7 (21)	15 (21)
	Factory, business, or service industry employee	16 (43)	10 (29)	26 (37)
	Farmer	12 (32)	8 (24)	20 (28)
	Student	1 (3)	6 (18)	7 (10)
	Retired	0 (0)	2 (6)	2 (3)
	Unemployed, able to work	0 (0)	1 (3)	1 (1)
**Marital status, n (%)**				
	Single	4 (11)	9 (27)	13 (18)
	Married	33 (89)	25 (74)	58 (82)
Cell phone can access the internet, n (%)		26 (72)	29 (85)	55 (79)
**Frequency of checking text messages, n (%)**				
	Immediately as they are delivered	20 (56)	17 (50)	37 (53)
	About every hour during the day	2 (6)	1 (3)	3 (4)
	At least 4 times a day, but not every hour	4 (11)	3 (9)	7 (10)
	2-3 times a day	7 (19)	6 (18)	13 (19)
	Once a day	1 (3)	1 (3)	2 (3)
	Less than once a day	2 (6)	4 (12)	6 (9)
	Never	1 (3)	0 (0)	1(1)
Daily cigarette smokers, n (%)		34 (92)	32 (94)	66 (93)
Number of cigarettes per day, mean (range)		12 (2-25)	15 (1-40)	13 (1-40)
**Nicotine dependence, n (%)**				
	Low	3 (8)	7 (21)	10 (14)
	Low to moderate	11 (30)	5 (15)	16 (23)
	Moderate	6 (16)	6 (18)	12 (17)
	High	17 (46)	16 (47)	33 (47)

### Qualitative Results

The major themes were smoking triggers, barriers to quitting and reasons for not wanting to quit, facilitators for quitting, perceived usefulness of text messaging for smoking cessation, suggestions for text message content, frequency and timing of message delivery, text messaging program duration, interactivity of text messages, perceptions about mindfulness and mindfulness-based methods for smoking cessation, and suggestions for incorporating mindfulness into smoking cessation programs.

#### Smoking Triggers

Smoking triggers were diverse among smokers in both groups. Most participants reported that they often wanted to smoke when they experienced stress or needed to concentrate or fight against sleepiness. In addition, many smokers indicated wanting to smoke when drinking alcohol, tea, or coffee by themselves or with friends at parties or coffee shops. Other common triggers included seeing someone smoking, seeing smoking friends, or being offered cigarettes. Some less common smoking triggers included cold weather and feeling bored:

I usually smoke when I need to concentrate on work, when I’m stressed, when seeing others smoke, it increases my craving and I want to smoke like them.

33 years, preparation stage

The first reason for smoking for me is feeling sad, staying up late, or because of work, maybe meeting with a friend and seeing him smoke also makes me want to smoke. Coming to a wedding or a funeral and meet so many of my friends there, I smoke, too.

48 years, contemplation/precontemplation stage

#### Reasons for Not Wanting to Quit (Among Smokers Without the Intention to Quit)

Believing that quitting is very hard or almost impossible was the most common reason for not wanting to quit among smokers who were in the contemplation or precontemplation stages. Many smokers reported that they did not want to quit because they needed to smoke to cope with stress/pressure from work, concentrate, or fight against sleepiness when working during night shifts. Other barriers to quitting included having friends who smoke and the availability of cigarettes:

Being addicted already, there must be a chance or a drug that could decrease the desire, the craving; therefore, giving up consequently. Just quit, how to quit? Quit, you cannot.

46 years, contemplation/precontemplation stage

I used to have the intention to quit smoking, two times, but haven’t been able to make it successfully because after quitting, I kept returning to the cigarettes when working at night. I also don’t know the ways as to how to quit smoking, how to make me less want to smoke. My aspiration for quitting smoking is still there, but I don’t know how to achieve that, so I keep smoking.

58 years, contemplation/precontemplation stage

#### Barriers to Quitting (Among Smokers Intending to Quit)

Among smokers with intentions to quit, a common barrier to quitting was frequently encountering other smokers or smoking friends (eg, seeing someone smoking, smelling smoke, receiving cigarette offers), which made it hard to control their cravings. Others found that the inability to cope with nicotine withdrawal symptoms was a barrier to quitting smoking, including bad or bland taste in their mouth, a feeling of missing something, concerns about gaining weight, or not being able to concentrate. In addition, some participants reported that they needed to smoke when they felt stressed or sad. Some smokers perceived their smoking as a long-time habit that was difficult to give up:

Actually, I want to quit smoking for a long time. It is hard. If I quit, I am not able to hang out with my friends. It will be a hard time because I cannot join my drinking buddies. It is only possible when I am alone or at home. It is impossible to quit if there are people smoking around me.

49 years, preparation stage

#### Facilitators for Quitting

Many smokers in both groups indicated that concerns about the harmful effects of smoking on themselves and their wives, children, or other people around them could motivate them to quit smoking. Complaints and encouragement from family members, especially wives and children, or friends and colleagues were also perceived as facilitators for quitting for many smokers, regardless of their stage of change. Other facilitators included medication to help cope with cravings, being in nonsmoking environments (eg, banning smoking in public places, staying away from other smokers), having a friend to quit smoking together, and being told by a doctor that they have a serious disease as a result of smoking (eg, cancer). Some smokers in both groups indicated that nothing could help them quit smoking, and it all depended on their own will or determination:

My motivation is that I worry about the health problem of myself and people around; I tend to actively participate in social and sports activities to cut down on stress in order to overcome the desire of smoking cigarettes.

33 years, preparation stage

First, brothers, peers, friends, and neighbour’s encouragement. For example, your children said that you have to stop smoking, this and that - for example, it is also bad for health. Secondly, for instance, there is a drug in order to... just like I said - to help quit smoking - to reduce the craving, if you are determined, it may be combined, then you can quit; if somebody is just thinking about to quit, it will be difficult. Self-quitting is hard. I feel it pretty hard.

46 years, contemplation/precontemplation stage

#### Level of Interest in SMS Text Messaging Smoking Cessation Programs

Overall, the majority of smokers with intentions to quit indicated a high interest in receiving text messages that support smoking cessation. They believed that messages that encourage them to quit or provide them with more information could be helpful. However, many smokers without intentions to quit said that text messaging would not be helpful because they perceived quitting to be impossible or believed that their ability to quit only depended on their determination.

#### Concerns About SMS Text Messaging Smoking Cessation Interventions

When asked about their concerns about text messaging to quit smoking, some smokers thought that there had already been many educational campaigns about smoking (eg, communication campaigns about the harms of smoking via radio, television, newspapers, posters, billboards, or label warnings with scare tactics), and therefore the text messaging program would not be effective if it were similar to these other communication programs. In addition, many smokers worried about receiving too many messages, which could be irritating as they already received too many spam messages. There were also concerns that a text messaging program might not be suitable for older people who might be less familiar with text messaging or have difficulty reading messages of a small font size. Finally, participants who owned basic cell phones with small black-and-white screens reported being less interested in reading messages:

This, I think, has been communicated regularly already, for a long time until now, the communication about quitting smoking has been conducted on every channel, from the cover of the cigarette pack and so on, so text messages will not work.

40 years, contemplation/precontemplation stage

First of all, the problem is that this text message thing is not really feasible. It’s only feasible for people who usually read messages, but a lot of people don’t usually do that. Such as old people, people with poor eyesight; not being able to see the texts clearly, they won’t read the messages, then there’s no effect.

44 years, contemplation/precontemplation stage

#### Suggestions for Message Content

When asked about the content of messages that would help to encourage smokers to think or actually quit smoking, the negative effects of smoking was the most common suggestion of smokers in both groups. Many believed that messages should mention the harmful effects to smokers and their family, and some smokers thought that they should include concrete evidence or specific examples (eg, the number of people who die because of smoking or the number of people who died from cancer caused by smoking, how smoking affects health, diseases caused by smoking). A few smokers suggested that fear-inducing content about the effects of smoking would motivate them to quit:

We should say that smoking affects every aspect of a person’s life, health, and people around them. The wording should make them frightened. It has to involve family, only then they will think that the smoke can affect their wife, children, and the people around them.

53 years, preparation stage

In the content, we can refer to health warnings, then warnings about the effects smoking might have on people around, their closest people like their parents, wife, and children. My wife also doesn’t like me to smoke, because when I smoke, my breath stinks, and she doesn’t like that.

48 years, contemplation/ precontemplation stage

Smokers in both groups suggested that text messages should provide quitting tips and encouragement, including strategies for coping with cravings. Some participants thought that smokers already knew about the harmful effects or health risks of smoking, and therefore, the messages should not focus too much on that issue but further highlight the effects of smoking on other family members (eg, wife and children). Other less common content suggestions included information about smoking cessation medication, the impact of smoking on finances, and the benefits of quitting to smokers:

I think the content of the messages should refer to how we can reduce the stress, how to deal with the craving for cigarettes when you’re upset and want to smoke, like instructing people what to do in order to achieve the aforementioned goals, taking a bath or meditation, since each person has their own circumstances and it’s not like everyone can sit whenever they want to.

46 years, preparation stage

Men are the pillars of family. First, we worry about our health, how we can work to earn money to support our family, wife, and children. You can deliver messages about the effects on our health if we couldn’t quit, it’ll affect not only our health but also your family.

29 years, contemplation/ precontemplation stage

Some smokers indicated that including photos in the text messaging program, images showing diseases caused by smoking (similar to graphic warning labels on cigarette packages), could be helpful. Images could also show successful cases of quitting smoking or the happiness of a family with a former smoker. Some smokers suggested that the program could send videos about smokers who are suffering or about the health consequences of smoking:

The content of the images and videos will be similar to the messages you want to deliver. If you want to propagate about the fact smoking can cause lung cancer, you should use images of a lung cancer case to shock people. We can tell how horrible it is after just one glance at that. Or use a video with scenes of a patient trying to get this disease cured or add a report or conversation between a doctor and a patient who has lung cancer because of smoking. I think that way, they will be impacted more directly.

41 years, contemplation/precontemplation stage

#### Frequency of Message Delivery

Smokers with intentions to quit smoking wanted to receive messages more frequently than smokers without the intention to quit. Most smokers with intentions to quit said that receiving 1-2 messages per day would be appropriate if the content of the messages is diverse. Some smokers in this group suggested having 3 messages per day. Only one smoker said that one message every 4 days would be enough. Some smokers suggested that the frequency could be decreased over time (eg, 2-3 messages per day in the first month and then decrease to 1 per day or 1 per 2 days in the subsequent months):

One message in the morning for the whole day. If three messages have the same content and purpose, people only need to read one then understand. For example, you can send three messages with different contents. Unless we send three messages with different information, one message is enough. This is my personal opinion.

36 years, preparation stage

One message per day in the evening, but the content should be diverse. Many messages have such poor content. If it comes during work hours, I’d be mad.

38 years, preparation stage

In the group of smokers who did not have the intention to quit, the suggested frequency varied from 4 messages per day to 3 messages per month. The most common suggestion was sending messages every day (1 to 3 messages per day), followed by sending 2 to 3 messages per week:

I think the number of messages should decrease gradually by months, one message every two days in the morning at first, then in the second month it will be one to two messages per week. The program should last for about one year and the messages should be sent in the early morning.

29 years, contemplation/ precontemplation stage

#### Timing for Message Delivery

Most smokers in both groups suggested that they would prefer receiving messages in the early morning (from 6 AM to 8 AM). Evening time (after dinner and before bedtime, 7 PM to 9 PM) was also suggested by many smokers. Some smokers mentioned that they often smoked in the early morning after waking up; therefore, sending messages at that time would help remind and encourage them to stay smoke-free. Sending messages in the evenings about the negative effects of smoking would make them think more about quitting. Other suggestions included sending messages after working hours in the morning and afternoon, when smokers often gather to smoke together, or after lunch, before working hours in the afternoon:

I often smoke in the early morning. So, if you send out messages at six or half past six a.m., and I receive the message while I’m smoking, maybe I can smoke less in the morning.

38 years, preparation stage

The most effective, I think, the moment is in the morning, and before bedtime, the message notifying me about the effect [of smoking] is usually in the evening. Imagine, when I have a big cough, the message comes, I find it helpful and this, the harmful cigarette, is so true.

48 years, contemplation/ precontemplation stage

#### Duration of the Text Messaging Program

Among those with intentions to quit, the majority thought that the program should last for about 3-6 months. Many smokers thought that it should last for a year to help them quit smoking. Some smokers thought that it should last for 1 month. Only a few smokers indicated that the program should last for just 1 week to 10 days:

I think we should send one or two messages per day, in about three months. That’s enough time for people to absorb everything.

55 years, preparation stage

I think there should be two messages a day at an early time. The first period of time is about three months, and we send two a day. After three to six months, we send one message a day or one per two days. After they could understand, then we should also decrease. But in general, the intervention should last for a year.

32 years, preparation stage

Among smokers who had no intention to quit, most thought that the program should last for a year to help them quit smoking effectively. The second most common suggestion was 3-6 months. The shortest duration was 1 month but was only suggested by a few smokers in this group:

It should last for about one year, in my opinion, it’s up to [individual’s] attitude, it’s no difference even for longer time.

23 years, contemplation/ precontemplation stage

#### Interactive Messages

Almost all smokers in both groups thought that interactive messages would be helpful for quitting. Only a few smokers who had no intention to quit thought that sending/receiving interactive messages would not be helpful to quit smoking:

That [interactive message] will be more helpful, more helpful. And so, when you are in the stage of craving for a cigarette, but now you wrote a message to send to see how to reduce the craving.

57 years, preparation stage

As far as I understand, two-way communication is always much better. My point of view is, I strongly agree, for people to have two-way information, there are things that we need advice, if we need to confer directly, then ask immediately.

52 years, contemplation/precontemplation stage

One smoker suggested that there should be a keyword list for smokers. When asked about suggestions for the keywords to be included on the list, smokers found it hard to give suggestions. However, some smokers suggested that they wanted to be able to request help via text messaging when they had cravings (eg, while drinking alcohol or feeling sad) or nicotine withdrawal symptoms. It was suggested that the program could consider developing a list of keywords based on the list of nicotine withdrawal symptoms in addition to basic keywords such as stress, crave, or slip:

My idea is that the switchboard could send a list, a list of motivated points to get rid of cigarettes. Example A: Stress, B: Tired, C: problems related to the mental/physiological issues in order to give up cigarettes. Then the receiver will be aware of these issues. According to that message, people could request for advice.

33 years, preparation stage

#### Perceptions About Mindfulness

After listening to the explanation about the concept of mindfulness and having a short mindful breathing practice, most participants perceived mindfulness as a method that helps to relax or reduce stress or tension and calm the mind, or pay attention or focus on doing one particular thing to forget cravings or other things. Some other smokers related mindfulness as a method to help them to establish a better habit to replace smoking, a method that helps focus on reality and avoid thinking about nonsense or a method that helps people forget their cravings. Some other smokers understood mindfulness as a meditation method or yoga practice:

It is the approach that helps you avoid stress, and relax your mind, with comfortable mind you don’t think much about other things, you are not affected and stimulated.

32 years, preparation stage

As far as I think, this is to create for you a habit to focus on one thing to forget other things, which means mindfulness, it is now you pay your attention to what is happening and ignore the other things, I mean that is mindfulness. I think it is also good; there is no problem then.

54 years, contemplation/ precontemplation stage

#### Mindfulness for Smoking Cessation

When the participants were asked about including mindfulness as a part of the program, almost all smokers thought that mindfulness would be very helpful for smoking cessation. They thought that it would help them overcome cravings, think less about smoking, or reduce stress, which is a common trigger for smoking:

This method helps us relax, make our mind comfortable, undisturbed, and not to worry about anything. No irritation, no thought about smoking in our mind, no feelings of craving. I think this is a wonderful method. It’d be extremely good if we can apply this method. Just sitting like that and totally relax, not thinking about anything. I think it’s something like that.

55 years, preparation stage

Before the discussion, everyone usually says that it’s the stress from work, irritation, and sorrow that make them stressed, now that this mindfulness can help reduce their stress and eliminate all those three factors. After erasing them, of course, we can smoke less, I think it’s really good.

58 years, contemplation/precontemplation stage

However, a few smokers thought mindfulness was not helpful for smoking cessation or had concerns about applying mindfulness-based approaches. One of these concerns was that practicing mindfulness might be difficult, especially for impatient people or smokers who have been smoking for a long time. In addition, longer formal mindfulness practices that take more time (eg, long bouts of sitting meditation or yoga) might not be feasible for everyone. Some smokers indicated that they would not be interested in mindfulness if it were described as a religious practice. Smokers who perceived the need to smoke as a way to stay alert or cope with fatigue said that mindfulness practices might make them feel sleepier.

#### Suggestions for Incorporating Mindfulness Into Smoking Cessation Programs

Many smokers thought that mindfulness should be incorporated into smoking cessation programs using videos (eg, posted on websites such as YouTube or Facebook) together with some written instructions. Mindfulness could be practiced through simple activities, such as walking, playing sports, or gardening, as formal meditation practice might not be feasible for every smoker. Some smokers were concerned that a program that only uses text messages (without photos or videos) to teach mindfulness would not be sufficient, as each message has a limited number of characters, and mindfulness is a relatively new concept that might be difficult to understand at first. One strategy might be to encourage smokers to practice mindfulness (eg, suggestions for focusing on the present moment and coping with cravings without automatically reacting by smoking), without necessarily using the term *mindfulness*, which could be confusing. Some smokers suggested that in-person instruction and guidance would be helpful for learning about mindfulness.

## Discussion

### Principal Findings

This study examined perceived smoking triggers, barriers and facilitators to quitting smoking, and perceptions about SMS text messaging and mindfulness-based smoking cessation methods among Vietnamese male smokers. Several important findings emerged: first, the majority of Vietnamese smokers with intentions to quit in our study, but only a few of those without the intention to quit, expressed interest in the SMS text messaging program. Second, regardless of their intentions to quit, participants indicated that the message content should be diverse to avoid repetition. They suggested that the messages could include (1) information about the harmful effects of smoking not only on smokers but also on the people around them (eg, wives and children), (2) facts about the consequences of smoking with specific data or evidence, and (3) positive encouraging messages, particularly effective tips for coping with cravings. In addition, each message should be concise, use language familiar to smokers, and avoid ambiguous or confusing words. This is particularly important because standard text messages that are compatible with all types of cell phones in Vietnam do not include accents, and without accents, the meaning of words in the Vietnamese language can sometimes be misunderstood. Third, many smokers preferred that messages be sent every day, with 1 to 2 messages per day, preferably in the early morning and evening. In addition, many smokers suggested that a text messaging–based smoking cessation program should last for approximately 3 months. Finally, many Vietnamese smokers were interested in the application of mindfulness in smoking cessation. Most participants perceived that mindfulness could potentially help with quitting, but some suggested that additional resources may also be needed to supplement teaching mindfulness through text messages.

### Comparison With Prior Work

The most commonly perceived smoking triggers were consistent with those reported in studies with other populations of adult smokers, including stress [27-30] and drinking alcohol [31]. In addition, beverages such as tea or coffee, popular drinks of Vietnamese people, can be potent triggers for many Vietnamese smokers. Smoking cessation interventions should encourage smokers to think about their own personal triggers, which might include culturally specific triggers (eg, social events where smoking is common among Vietnamese men), and plan ways to avoid and/or cope with them.

Among smokers who had no intention to quit, the most common reason was the perception that quitting is very difficult or impossible. This perception might be a result of their failure in their past attempts to quit or because of the lack of effective methods to deal with cravings and withdrawal symptoms. Other quitting barriers found in both groups of smokers were similar to those reported in previous studies, such as perceiving the need to smoke for stress management, concentration, cravings, and withdrawal symptoms [32]. Smokers noted that an awareness of the harmful effects of smoking was an important motivator for quitting, which is similar to other studies, highlighting that an awareness of the negative effects of smoking might lay the foundation for progress toward smoking cessation [33]. In addition, smokers perceived that complaints and encouragement from other people would also help motivate them to quit. Consequently, smoking cessation interventions may need to focus on increasing the self-efficacy of smokers who have no intention to quit and provide them with strategies to cope with cravings, at the same time, encouraging smokers who have the intention to quit to elicit support from their family members or friends.

With regard to the development of an SMS text messaging smoking cessation program, we found that strategies or tips for managing their cravings and motivational or encouraging messages were the most common desired content among the smokers in our study. This is consistent with the findings from previous studies on adult smokers in the United States [34,35]. In addition, Vietnamese smokers in our study valued content about the harmful effects of smoking not only on smokers themselves but also for their family members. Smokers also indicated a preference for specific evidence, facts, or numbers that could motivate them to quit and remain smoke-free. The inclusion of photos, videos, and interactive functions was valued by most smokers. However, despite the ubiquitous interest in having photos or video links included in the messages, these formats might only be compatible with smartphones. Interestingly, we found that the use of *scare tactics* suggested by smokers in the study by Bock et al [34] might not work for Vietnamese smokers as the smokers in our study indicated that health communication programs and warning labels on tobacco packs already used such tactics, and therefore additional messaging might not have strong effects. Another difference between our study and previous qualitative studies of text messaging for smoking cessation (most of which were conducted in the United States or other high-income countries) is that most smokers in our study did not want to receive more than 2 messages per day. Studies indicate that smokers in developed countries may prefer to receive more messages, even 5 to 6 messages per day around their quit date [12,34,35]. This difference might be explained by the large number of spam messages sent to cell phone subscribers in Vietnam, which made Vietnamese cell phone users reluctant to receive text messages. The 3-month duration of the text messaging program preferred by smokers in our study is consistent with that reported by Bock et al [34] and aligned with implemented trials [12].

After trying a brief mindful breathing practice, participants described mindfulness as a method that could help them relax, reduce stress or tension, calm their mind, or improve concentration. Overall, participants had a positive attitude toward mindfulness, and they believed that this method would be helpful to quit smoking. However, it may be important to clarify with this population that although mindfulness can help reduce stress and anxiety [36,37], it is not the same thing as relaxation. In fact, given that mindfulness involves nonjudgmental attention to events occurring in the present moment, practicing mindfulness can be very uncomfortable (eg, when noticing sensations of craving, pain, or unpleasant emotions). In addition, participants noted that text messages might not be sufficient for teaching mindfulness, given that the concept of mindfulness was relatively new to most Vietnamese smokers in this study. Moreover, even in intensive in-person training programs, participants typically do not practice mindfulness on their own as much as instructed [38]. Text messages could be a useful modality for reminding people to practice mindfulness regularly, but it might be optimal to combine text messaging with web-based, telephone-based, or in-person training. For example, text messages have recently been combined with in-person mindfulness-based treatment for smoking cessation in the United States. It might also be helpful to describe mindfulness practices in clear, understandable ways (eg, slowing down to pay attention, focusing on your breathing), without using the term mindfulness, if that causes confusion for some people.

### Limitations and Conclusions

This study has several limitations. Our study included only Vietnamese male smokers; therefore, the results may not generalize to smoking cessation programs aimed at female smokers or those in other geographic areas. In addition, this study examined the ideas smokers have about receiving text messages for smoking cessation rather than their actual experiences of receiving sample text messages. An important future direction will be to collect qualitative data after Vietnamese smokers have the opportunity to receive a text messaging intervention. However, this study is strengthened by a relatively large sample size for qualitative research; investigation of themes separately by smoking stage of change; examination of mindfulness as a relatively novel potential strategy for smoking cessation in Vietnam, and inclusion of smokers from diverse backgrounds based on age, education level, working status, and place of residence.

Our findings provide important insights into smoking cessation programs for male smokers in Vietnam, a country with a high prevalence of smoking and related morbidity and mortality among men, but low accessibility to smoking cessation treatment. Overall, participants (especially those with intentions to quit smoking) were interested in SMS text messaging as a method to help them quit smoking. They suggested that the messages should be nonrepetitive and have diverse content, including concrete evidence and statistics about the negative effects of smoking for both smokers and their family members. In terms of message frequency, delivering 2 messages per day either during early morning or evening was acceptable to most participants, although an individually tailored delivery of messages catering to specific individual needs would be even better. Mindfulness training was viewed favorably by Vietnamese smokers as a potential solution to help them quit; however, some suggested that videos and/or in-person training may also be needed to supplement teaching mindfulness through text messages.

## Abbreviations

mHealth: mobile health
